# Policy-mix and SME innovation: Evidence from China

**DOI:** 10.1371/journal.pone.0319080

**Published:** 2025-02-25

**Authors:** Kai Zhao, Haonan Shan, Yu Gao

**Affiliations:** School of Economics, Qingdao University, Qingdao, Shandong Province, China; University of Almeria: Universidad de Almeria, SPAIN

## Abstract

The question of whether and how innovation policy can effectively influence innovation in small and medium-sized enterprises (SMEs) has received limited attention in academic research. This study takes a first step towards filling this gap by examining how innovation policy and policy mixes influence innovation in SMEs. This paper takes China’s National Equities Exchange and Quotations (NEEQ) listed enterprises from 2011 to 2020 as the research sample, and uses the Multi-Level Treatment Effect (MLTE) model to investigate the actual impact of different innovation policies on Small and Medium-sized Enterprise innovation and the heterogeneity of policy effects from the perspective of substantive and strategic innovation. It is found that innovation policies can obviously improve the innovation of SMEs, in particular the substantive innovation, and the effect of policy-mix in stimulating SME innovation is stronger than that of single innovation policy. SMEs that show “strong motivation” and “high ability” in innovation are more likely to be favored by relevant government agencies, and have a greater probability of becoming the implementation targets of innovation policies. As far as single innovation policies are concerned, government subsidy is better than tax incentive for high-tech SMEs, while tax incentive has a stronger role in promoting innovation than government subsidy for non-high-tech SMEs. By illuminating these differentiated impacts and the conditions under which innovation policies are most effective, this work not only advances our fundamental understanding of policy-driven innovation ecosystems but also offers actionable guidance to policymakers seeking to optimize the allocation of support to foster transformative innovation in the SME sector.

## 1 Introduction

Innovation is not only the first driving force leading development, but also a strategic support for building a modern economic system. Following a swift period of economic advancement and considerable scale enlargement built upon the utilization of factor inputs like resources, capital, and labor, China has transitioned into a phase termed as the “New Normal.” This era is marked by shifts in pace, enhancements in structure, and alterations in authority. Becoming the pivotal element for propelling and upholding enduring, top-notch economic progress, innovation has taken the forefront. Small and Medium-sized Enterprises (hereinafter referred to as SMEs), emerging as a fresh driving force in national economic and social progress, indisputably hold the reins for fostering innovation, ensuring steady growth, and averting potential risks. As per statistics released by the Ministry of Industry and Information Technology in China, the count of SMEs surpassed 30 million in 2019 [1]. These SMEs play a substantial role in the economic landscape, contributing over 50% of the overall tax revenue, over 60% of the Gross Domestic Product (GDP), over 70% of technological advancements, and over 80% of employment within the labor force [2].

Although China’s SMEs are booming, most of them still face problems such as relatively backward process equipment, lack of innovative talents and resources in the process of innovation, and these problems have become constraints to the sustainable innovation and development of SMEs [3,4].Recently, China has successively formulated and promulgated a series of important policies, such as “Several Opinions on Deepening the Reform of System and Mechanism and Accelerating the Implementation of Innovation-Driven Development Strategy”, “12th Five-Year Plan for the Growth of Small and Medium-sized Enterprises”, and “Several Opinions on Further Promoting the Innovation and Development of Small and Medium-sized Enterprises of Science and Technology”, to support the innovation of SMEs and stimulate their innovation vitality and motivation through government subsidies and tax incentives [5–9]. However, as the formulation and promulgation of China’s innovation policies involve numerous institutions, and there is little cross-departmental and functional coordination, the situation of “policy crowding” often appears [10]. Taking the research sample of this study as an example, 23.96% of SMEs are supported by government subsidy, 26.14% supported by tax incentive, and SMEs supported by policy-mix accounted for 19.20% of the total sample.

In light of the disparate levels of policy support received by SMEs and the inherent challenges associated with the implementation of innovation policies, the research questions posed in this paper are as follows: Does the impact of innovation policies on SME innovation vary depending on the specific policy in question? What is the impact of innovation policy on the selection of enterprise innovation strategies? Does the integration of innovation policies yield superior outcomes compared to a single approach? Which SMEs are the most deserving of the focus of innovation policies? By studying the aforementioned questions, a foundation can be established for government departments to inform the formulation, adjustment, and allocation of resources in policy. This facilitates the formulation of policies that more accurately align with the needs of enterprises, thereby enhancing policy efficiency and effectiveness.

Integrating different innovation policies into a coherent econometric model framework has always been a challenge in policy evaluation research. This study aims to address that challenge. Therefore, this paper uses a multi-level treatment effect model (MLTE) to investigate the impact of government subsidies, tax incentives, and their combinations on SME innovation in the real world, based on a sample of enterprises listed in the China National SME Share Transfer System from 2011 to 2020. Compared with previous studies on SME innovation from a single perspective [11–13], this paper examines whether innovation policies have an impact on firms’ innovation strategy choices from both substantive and strategic innovation perspectives. The study finds that innovation policies can significantly improve SMEs’ innovation, especially substantive innovation, and that the incentive effect of policy combinations on SMEs’ innovation is stronger than that of single innovation policies. Second, previous studies have not addressed which firms deserve the focus of innovation policies [14,15]. This paper finds that SMEs that show “strong motivation” and “high ability” in innovation are more likely to be favored by relevant government agencies and more likely to become the target of innovation policy implementation. In addition, for SMEs in the eastern region, the policy mix is the optimal innovation policy to promote substantive and strategic innovation; for SMEs in the central and western regions, government subsidies and policy mix may be the optimal strategies to promote substantive innovation, while tax incentives and policy mix may both be the optimal strategies to promote strategic innovation. Finally, in terms of a single innovation policy, government subsidies are superior to tax incentives for high-tech SMEs, while tax incentives have a stronger effect on innovation than government subsidies for non-high-tech SMEs.

The primary contributions of this study are outlined below. Primarily, prevailing studies tend to focus on assessing the impact of innovation policies on SME innovation individually, overlooking the crucial aspect that SMEs might derive benefits from a spectrum of innovation policies concurrently. This oversight can introduce treatment biases, potentially undermining the validity of existing conclusions. Furthermore, issues arise from SMEs self-selecting into innovation policies and governmental agencies employing “picking-the-winner” strategies, resulting in endogeneity concerns [16]. To address these challenges, this study adopts the Multi-Level Treatment Effect (MLTE) model. This model is adept at pinpointing the optimal policy mix and methodically evaluating the tangible effects of varied innovation policies and their combinations on SME innovation in China. Importantly, it tackles endogeneity and selection bias issues effectively, ensuring a robust analysis. Secondly, the innovation efforts within SMEs might primarily serve as a managerial tactic, not necessarily aimed at significantly enhancing technological competitiveness but rather to secure specific advantages, often aligning with government directives. This study delves into a detailed analysis of how various innovation policies and their combinations influence the substantive and strategic innovation within SMEs, respectively. By exploring these dimensions, the study aims to unravel the intricate relationship between innovation policies and SME innovation from diverse perspectives.

The subsequent sections of this paper are organized as follows. Section 2 provides an overview of the literature review. Section 3 outlines the research design. Section 4 presents the data source and details the measurement of variables. Section 5 deliberates on the estimated results. Lastly, in Section 6, the study concludes and presents policy recommendations.

## 2 Literature review

### 2.1 SME and innovation

Various countries establish distinct definitions for SMEs [17]. For instance, in Europe, SMEs encompass enterprises with fewer than 250 employees, an annual turnover below 50 million euros, and/or an annual balance sheet total not exceeding 43 million euros. Belgium sets a cap at 100 employees, whereas Germany considers up to 225 employees [18]. In China, defining SMEs is intricate, with variations across different industries [19]. Despite the absence of a universally accepted definition of SMEs across countries, their significance is irrefutable. First, SMEs make a significant contribution to employment and economic development, which can enhance economic resilience and improve competitive advantage [20–22]Secondly, SMEs possess the agility and innovative potential [23,24] to play an indispensable role in the adoption of sustainable technologies and the promotion of sustainable development [25,26].

In addition to the aforementioned definition of SMEs and the study of their importance, academic research on SMEs has focused on several key areas, including their digital transformation [27,28], marketing strategies [29–31], financial performance [32,33] and others. A limited number of studies have examined innovation in SMEs. In the context of SMEs, innovation typically pertains to the development of a new or notably enhanced product, service, or process within an enterprise [34,35]. Innovation can be categorized into strategic innovation and substantive innovation. Strategic innovation involves expanding product lines or modifying existing platforms and products to meet market or customer needs through enhancements and extensions. It can also serve as a strategic approach aimed at satisfying governmental requirements by prioritizing the “quantity” and “pace” of innovation to pursue other objectives [36]. On the other hand, substantive innovation focuses on high-quality innovations geared towards advancing technological progress and enhancing the competitive edge of SMEs.

### 2.2 Innovation policies

Innovation policy is of significance at both the macro and micro levels. From a macroeconomic perspective, innovation policies can facilitate technological advancement and, in turn, help to sustain national competitiveness [37]. Innovation policies and education policies are mutually reinforcing and collectively contribute to economic growth [38]. Additionally, innovation policies can enhance a city’s green total factor productivity through the aggregation of talent and augmented scientific and technological expenditure [39]. At the micro level, innovation policies are typically classified into two categories: government subsidies and tax incentives. Government subsidies serve as *ex ante* incentives for innovation [40], while tax incentives function as *ex post* incentives [41,42]. Despite numerous studies exploring the relationship between innovation policies and innovation outcomes, empirical research has yet to yield consistent conclusions. Regarding government subsidies, empirical studies highlight the coexistence of various effects such as the crowding-in effect [43], crowding-out effect [44], non-linear effect [45], and dynamic effect [46–48]Similarly, there is no consensus on the relationship between tax incentives and innovation, with research findings suggesting incentive effects [49,50], inhibiting effects [51], and moderate interval effects [52].

### 2.3 Policy-mix and SME innovation

An innovation policy mix is defined as the collective implementation of a range of policies designed to facilitate technological advancement, industrial growth, and social transformation. This approach not only considers the role of individual policy tools, but also emphasises the importance of collaboration and the overall effectiveness of policies.

Research on the correlation between innovation policy-mix and SME innovation is relatively limited. Radas et al. [53] discovered that a policy-mix comprising government subsidies and tax incentives can foster innovation output among enterprises, based on data from Croatian SMEs. Conversely, Dumont [54] concluded that the combined implementation of government subsidies and tax incentives has a lesser impact on innovation compared to the use of a single innovation policy. Radicic and Pugh [55] conducted an analysis on SME data from 28 European countries and determined that policy-mixes at various levels (European and national) are beneficial for promoting SME innovation.

## 3 Research design

Actually, due to the obvious tendency of government subsidies, tax incentives and their combination implemented by the government to SMEs [56], for example, high-tech enterprises or enterprises that can create a large number of employment opportunities are more likely to get government support [57]. Therefore, whether SMEs can enjoy innovation policy support does not meet the requirement of random distribution. Besides, the existence of information asymmetry makes it difficult for the government to obtain the real information to judge whether the target SME should be supported or not, which makes the characteristics of SMEs (*i.e.*, enterprise scale, leverage ratio) affect the probability that SMEs get governmental support [58]. Therefore, it is imperative to address the selection bias stemming from the non-random distribution characteristics of innovation policies when assessing their impact on SME innovation. While Propensity Score Matching (PSM) and Endogenous Switching Regression (ESR) are remedies for selection bias, they necessitate treatment variables to be binary (dummy) in nature. To gauge the Average Treatment Effects on the Treated (ATT) across multiple treatment states within a “counterfactual” framework, it is crucial to establish the large-sample properties of Efficient-Influence-Function (EIF) estimators, Inverse-Probability Weighted (IPW) estimators, and other features of potential-outcome distributions [59]. By employing these estimators, known to be semiparametrically efficient under specific regularity conditions [60], a diverse array of treatment-effects estimators can be formulated alongside valid inference procedures for multivalued treatment effects. In this study, the Multi-Level Treatment Effect (MLTE) model is utilized to proficiently identify the optimal policy-mix and systematically evaluate the actual impact of various innovation policies and their combinations on SME innovation in China.

Consider the SME data with *n* observations in which each SME has been assigned one of J+1 possible treatment levels j=0,1,⋯,J. For each SME i=1,2,⋯,n, we observe the random vector $z_{i}={\left(y_{i},T_{i},{\text{x}}_{i}^{\prime }\right)}^{\prime }$, where $y_{i}$ is the observed outcome of SME innovation, $T_{i}$ denotes the treatment level administered, and ${\text{x}}_{i}^{\prime }$ is a $k_{x}\times 1$ vector of covariates. In our study, j=0 if the SME is only supported by government subsidies; j=1 if the SME is only supported by tax incentives; j=2 if the SME is supported by both government subsidies and tax incentives. We define the indicator variables $d_{i}(j)=\text{l(}T_{i}=j\text{)}$, which take the value 1 if SME *i* received treatment *j* and otherwise 0. We use the classical potential-outcome framework in the context of multivalued treatment effects to describe the estimators of interest. This model distinguishes between the observed outcome $y_{i}$ and the J+1 potential outcomes $y_{i}\left(j\right)$ for each treatment level j=0,1,⋯,J. The observed explained variable is given by Equation (1).


$$y_{i}=d_{i}\left(0\right)y_{i}\left(0\right)+d_{i}\left(1\right)y_{i}\left(1\right)+\cdots +d_{i}\left(J\right)y_{i}\left(J\right)$$ (1)

where $\{y_{i}\left(0\right),y_{i}\left(1\right),\cdots ,y_{i}\left(J\right){\}}^{\prime }$ is an independent and identically distributed draw from $\{y\left(0\right),y\left(1\right),\ldots ,y\left(J\right){\}}^{\prime }$ for each SME i=1,2,⋯,n. The distribution of each $y\left(J\right)$ is the distribution of the explained variable that would occur if SME were given treatment level *j*; it is known as the potential-outcome distribution of treatment level *j*.

In the realm of causal inference, adherence to the conditions of random distribution necessitates that the multi-level treatment effect model satisfies two pivotal assumptions: the Conditional Independence Assumption (CIA) and the Overlap Assumption (OA). CIA requires that the distribution of each potential-outcome $y\left(j\right)$ is independent of the random treatment variable $d\left(j\right)$ given covariate ${\text{x}}_{i}^{\prime }$, in other words, y(j)⊥d(j)|x. OA requires that the probability that the SMEs are arranged in any treatment state based on covariate is positive, namely $p_{j}(\text{x})=\mathrm{Pr}(T=j|\text{x) }>\text{ 0}$. In line with the aforementioned assumptions, the functional form representing the conditional expectation value of SME innovation can be articulated as follows:


$$\text{Ε}\left[y\left(j\right)|\text{x}\right]=\text{Ε}\left[y_{i}|T_{i}=j,\text{x}\right]=\beta_{0j}+\text{x}\beta_{\text{1}j}$$ (2)

The General Propensity Score (GPS) is used to calculate the Inverse-Probability Weighted (IPW) of the observed values of covariates in each treatment level $T_{i}$, so as to ensure the balance among different treatment levels [61]. Referring to the ideas of Cattaneo [59] and Cattaneo et al. [60], the Average Treatment Effect on the Treated (ATT) when the treatment level changes from $T_{i}$ to *k* (k∈{0,1,⋯,J}) can be estimated, specifically


$$ATT_{jk}=\left({\hat{\beta }}_{0j}-{\hat{\beta }}_{0k}\right)+\frac{1}{n}\sum_{i=1}^{n}x_{i}\left({\hat{\beta }}_{1j}-{\hat{\beta }}_{1k}\right)$$ (3)

## 4 Research Data

### 4.1 Data source

The data of listed enterprises in National Equities Exchange and Quotations (It is commonly known as “New Third Board”) market from 2011 to 2020 is selected as the research sample to analyze the impact of innovation policies on SME innovation. There are three main reasons. First, NEEQ contains many SMEs, and these SMEs are all over China’s provinces and cities, which is conducive to examining the effects of innovation policies on SME innovation from different levels and scopes. Second, NEEQ listed enterprises can provide externally audited financial information, which is reliable. Third, the patent application data of NEEQ listed enterprises can be directly obtained through the network platform, the data collection process is relatively simple. Specifically, the patent data of NEEQ listed enterprises come from the Baiten Patent Database. By matching the NEEQ listed enterprises, the patent application data of SMEs during 2011-2020 can be collected. The financial data, R&D data, and managerial data of NEEQ listed enterprises come from WIND database and China Stock Market & Accounting Research (CSMAR) database. The relevant data of the region where the NEEQ listed enterprises are located come from the “China City Statistical Yearbook”. To safeguard the validity and reliability of the data, the initial dataset undergoes the following processing steps: Large enterprises failing to meet SME criteria, as outlined in the “Statistical Measures for the Division of Large, Medium, and Small Enterprises” by the National Bureau of Statistics (detailed in Appendix A), are excluded. Observations from NEEQ listed enterprises displaying abnormal financial conditions are omitted. Observations related to financial and insurance enterprises are also excluded. Variables with a significant number of missing values are removed, and a Winsorize treatment is applied to continuous variables at the 1st and 99th percentiles. Following these data processing steps, the dataset is refined to incorporate 51,162 observations.

### 4.2 Variable measurement

Innovation measurement holds significant importance for policymakers and managers alike [62]. From the policymakers’ perspective, having clear and dependable indicators is crucial for formulating effective innovation policies, evaluating proposals for innovation projects, and monitoring the progress of funded initiatives [63]. For SME managers, demonstrating innovative capabilities can enhance their ability to attract funding from investors and government sources [64]. Patents serve as a valuable source of both qualitative and quantitative data on SME innovation, with online patent databases offering a convenient and immediate means of gauging innovation [65]. In this study, the number of patent applications is selected as the metric for measuring SME innovation [66]. Patent application data can quickly reflect current innovation trends and research and development priorities. Researchers, companies or governments can quickly obtain the latest information on technological development and innovation directions by tracking the number and types of patent applications. Therefore, patent application data provides a more timely snapshot of innovation activities. Building upon the concept introduced by Li and Zheng [67], SME innovation is further categorized into substantive innovation and strategic innovation. Substantive innovation, considered the cornerstone of SME innovation and a primary driver of SME growth, is assessed through the count of “Patent for Invention” filings by SMEs (Subs_inno). On the other hand, strategic innovation represents the tactics adopted by SMEs to align with governmental requirements and is quantified by the combined quantity of “Patent for Utility Model” and “Patent for Industrial Design” applications by SMEs, denoted as “Patent for Non-Invention” (Stra_inno).

The government enforces innovation policies through three channels: government subsidies, tax incentives, and a policy-mix approach. To capture whether SMEs receive government subsidies, we introduce the dummy variable Sub. When the SME is supported by government subsidy, Sub=1; otherwise Sub=0. Drawing on Hu and Wu [68], we use the intensity of tax incentives to gauge whether an SME benefits from tax incentives (refer to Table 1). In this paper, we first calculate the intensity of tax incentives (taxpre), where taxpre=1−ETR/TR, where ETR is the average effective tax rate, calculated as incometaxexpense/grossprofit and TR is the statutory tax rate, which is set to 25%. If taxpre > 0, then Tax=1, otherwise Tax=0. In cases where an SME simultaneously benefits from both government subsidies and tax incentives, the policy-mix dummy variable is set to 1. Based on these, innovation policy can be defined as the multivalued treatment variable *w*. Note that 15707 SME samples are not supported by any innovation policy (w=0), accounting for about 30.7% of the total observations; 12258 SME samples receive only government subsidies (w=1), accounting for 23.9% of the total samples; 13374 SME samples enjoy tax incentives (w=2), accounting for about 26.1%; 9823 SME samples enjoy both government subsidies and tax incentives (w=3), accounting for about 19.2%.

**Table 1:** Variable measurement.

Type	Name	Symbol	Measurement
**Outcome**	Innovation	Inno	Natural logarithm of (Number of patent applications + 1)
	Substantive innovation	Subs_inno	Natural logarithm of (Number of “Patent for Invention” + 1)
	Strategic innovation	Stra_inno	Natural logarithm of (Sum of the number of “Patent for Utility Model” and “Patent for Industrial Design” + 1)
**Policy**	Government subsidy	Sub	If SME receiving R&D subsidies Sub=1, otherwise Sub=0.
	Tax incentive	Tax	If $\frac{1-ER}{TR}>1$, Tax=1; otherwise Tax=0. ER means “effective tax rate” namely $ER=\frac{incometaxexpense}{grossprofit}$. TR means legal tax rate, TR = 25% according to relevant tax regulations (Zhao et al., 2022).
	Policy-mix	Mix	If the SME enjoys both government subsidies and tax incentives (policy-mix), Mix=1, otherwise Mix=0.
**Treatment**	Innovation policy	*w*	Multivalued treatment variable. w=0 indicates that the SME does not enjoy the support of any innovation policy; w=1 indicates that the SME receive only government subsidies; w=2 indicates that the SME receive only tax incentives; w=3 indicates that the SME enjoy simultaneously government subsidies and tax incentives.
**Control**	Enterprise scale	Size	Natural logarithm of total assets of SME
	Asset-liability ratio	Lev	Ratio of liabilities to total assets at the end of year
	Enterprise growth	Grow	Growth rate of total assets
	Ratio of assets expenditure	Cap	Ratio of total cash paid for the purchase of fixed assets, intangible assets and other long-term assets to total assets at the end of year
	Fixed asset ratio	PPE	Ratio of total fixed assets to total assets
	Board independence	Indep	Ratio of the number of independent directors to the total number of board members
	GDP	GDP	Natural logarithm of regional GDP
	Population	Pop	Natural logarithm of regional population

In addition, drawing lessons from the existing studies [69], enterprise scale (Size), asset-liability ratio (Lev), enterprise growth (Grow), capital-expenditure ratio (Cap), fixed asset ratio (PPE), and board independence (Indep) are selected as control variables. Besides, we use the economic level (GDP) and population (Pop) of the city where the SME is located to control the regional factors. Table 1 collects the name, symbol and measurement of the above-mentioned variables. Table 2 reports the descriptive statistics of the main variables.

**Table 2:** Descriptive statistics.

Variable	Mean	Standard deviation	Min	Max	Skewness	Kurtosis	Observation
Inno	0.8034	1.0550	0	7.1449	1.0472	3.0876	51162
Subs_inno	0.4180	0.7368	0	6.2724	1.9189	6.6128	51162
Stra_inno	0.5873	0.9100	0	5.9736	1.3861	3.9794	51162
Sub	0.2396	0.4837	0	1	-0.5227	1.2732	51162
Tax	0.2614	0.4804	0	1	0.5768	1.3327	51162
Mix	0.1920	0.3382	0	1	1.0986	2.2069	51162
*w*	1.3493	1.1352	0	3	0.3499	1.7152	51162
Size	18.2204	1.1628	14.8630	23.7392	0.0426	2.8021	51162
Lev	0.4023	0.2097	0.0335	1.4287	0.2883	2.4893	51162
Grow	0.1132	0.4717	-4.5654	0.9715	-6.3151	59.8399	51162
Cap	0.2119	0.1859	0	0.8623	0.9127	3.1001	51162
PPE	0.1629	0.1583	0	0.6973	1.1048	3.5708	51162
Indep	0.0189	0.0768	0	0.3861	3.9707	17.2013	51162
GDP	18.2233	0.9918	15.5913	19.8942	-0.5836	2.3749	51162
Pop	6.7943	0.6683	4.7113	8.5362	-0.4885	2.9946	51162

## 5 Results

### 5.1 Influence of different innovation policies on SME innovation

We employ the Multi-Level Treatment Effects (MLTE) model based on Generalized Propensity Scores (GPS) to assess the influence of various innovation policies on SME innovation. In Table 3 (lines 1–3), the estimated Average Treatment Effects on the Treated (ATTs) are presented for each of the three potential outcome distributions of the outcome variables (Inno, Subs_inno, and Stra_inno). The results suggest that the ATTs from the potential outcome distributions rise with the treatment level [60]. SMEs that benefit from innovation policies exhibit greater incentives to innovate compared to those without any support from such policies.

**Table 3:** Estimated ATEs of different innovation policies.

Variable	Inno		Subs_inno		Stra_inno	
	ATT	S.E.	ATT	S.E.	ATT	S.E.
Sub	0.1218***	0.0121	0.0751***	0.0086	0.0818***	0.0104
Tax	0.1229***	0.0161	0.0768***	0.0119	0.0942***	0.0146
Mix	0.3226***	0.0152	0.1928***	0.0101	0.2275***	0.0129
Tax Vs. Sub	0.0011	0.0162	0.0018	0.0115	0.0124	0.0140
Mix Vs. Sub	0.2007***	0.0144	0.1177***	0.0095	0.1457***	0.0122
Mix Vs. Tax	0.1996***	0.0186	0.1159***	0.0127	0.1332***	0.0158
Observations	51162		51162		51162	

**Note:**

***,

**, and

*  represent the significance levels at 1%, 5%, and 10%, respectively; S.E. represents Standard Error.

As far as single innovation policies are concerned, the outputs in line 4 of Table 3 report that the estimated ATTs of going from class-type 1 (w=1) to class-type 2 (w=2) are 0.0011, 0.0018, and 0.0124 respectively. These estimated values are not statistically significant, indicating that there is no obvious difference between government subsidies and tax incentives in promoting SME innovation, substantive innovation, and strategic innovation. The outputs in line 5-6 of Table 3 show the estimated ATTs of policy-mix level w=3 versus the single innovation policy level (w=2 or w=1). By comparing the effects of innovation policy-mix and single innovation policy on SME innovation, it is found that innovation policy-mix is always better than single innovation policy in terms of substantive innovation and strategic innovation.

### 5.2 Robustness check

As the innovation policy cannot be implemented according to the requirements of the evaluation, it is necessary to further test the applicability of the evaluation method and the reliability of the results. The applicability of the model and the reliability of the estimated results are checked by Overlap Assumption (OA) test and increasing control variables.

#### 5.2.1 *OA test.*

**Fig 1:**
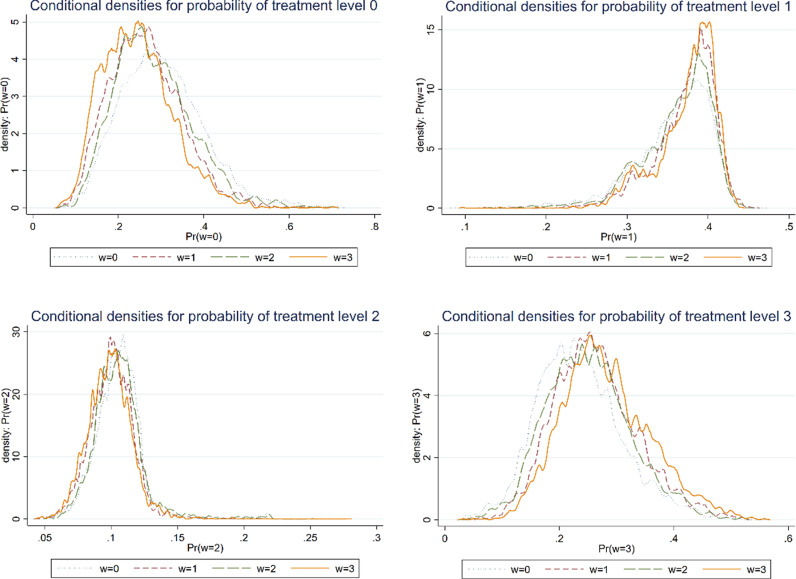
Conditional densities for probability of different treatment levels.

Meeting the OA is a prerequisite for analysis by multilevel treatment effect model. In line with [70], we utilize overlap plots to identify any potential problematic cases. Fig 1 illustrates the results of OA test. It is confirmed that the conditional densities for probability of treatment levels ($w=\left(0,1,2,3\right)$) are all greater than 0 and less than 1. As Fig 1 makes plain that OA test exhibits very good overlap, the applicability of the model estimation is verified.

#### 5.2.2 *Increasing control variables.*

The innovation of SMEs is not only the result of unilateral R&D resources investment by SMEs, but also may be closely related to the city where the SMEs are located. Upon revisiting the influence of various innovation policies on SME innovation and augmenting the control variables (GDP and Pop), the outcomes in Table 4 exhibit no discernible variance compared to the benchmark results in Table 3. This consistency reaffirms the reliability of the estimated findings.

**Table 4:** Robustness check: increasing control variables.

Variable	Inno		Subs_inno		Stra_inno	
	ATT	S.E.	ATT	S.E.	ATT	S.E.
Sub	0.1287***	0.0121	0.0811***	0.0086	0.0834***	0.0105
Tax	0.1293***	0.0170	0.0822***	0.0120	0.0958***	0.0147
Mix	0.3462***	0.0149	0.2093***	0.0101	0.2434***	0.0126
Tax Vs. Sub	0.0005	0.0163	0.0011	0.0116	0.0123	0.0141
Mix Vs. Sub	0.2174***	0.0142	0.1281***	0.0096	0.1599***	0.0118
Mix Vs. Tax	0.2169***	0.0185	0.1270***	0.0128	0.1476***	0.0157
Observations	51162		51162		51162	

**Note:**

***,

**, and

*represent the significance levels at 1%, 5%, and 10%, respectively; S.E. represents Standard Error.

### 5.3 Selection of target SMEs for different innovation policies

Table 5 explains how do government select the target SMEs for different innovation policies. On the whole, factors such as enterprise scale and leverage ratio can influence the government’s choice of target SMEs. In particular, a greater SME scale and a reduced leverage ratio correspond to an increased likelihood of benefiting from innovation policy support. Broadly speaking, large-scale SMEs possess robust production capabilities and tend to propel their progress through innovation [71]. On the other hand, SMEs with low leverage ratios can sustain ongoing research and development investments, fostering stronger innovation capabilities compared to high-leverage-ratio SMEs [72]. SMEs that show “strong motivation” and “high ability” in innovation are more likely to be favored by relevant government agencies, and have a greater probability of becoming the implementation targets of innovation policies.

**Table 5:** Target selection of innovation policies.

	w=0	w=1	w=2	w=3
Size	—	0.3294*** (0.0112)	0.1546*** (0.0158)	0.5137*** (0.0124)
Lev	—	-1.3490*** (0.0558)	-0.5936*** (0.0786)	-1.7659*** (0.0625)
Grow	—	0.0909*** (0.0248)	-0.1171*** (0.0283)	-0.0285 (0.0279)
Cap	—	0.1325 (0.1472)	0.1451 (0.2072)	0.4882*** (0.1578)
PPE	—	0.1851 (0.1718)	-0.2941 (0.2436)	-0.4168 (0.4850)
Indep	—	0.8232*** (0.1811)	1.2593*** (0.2267)	0.3309 (0.2766)
*Constant*	—	-5.1610*** (0.1950)	-3.5064*** (0.2764)	-8.7300*** (0.2175)
Observations	15707	12258	13374	9823

**Note:**

***,

**, and

*  represent the significance levels at 1%, 5%, and 10%, respectively; standard error is presented in parentheses.

Furthermore, as far as single innovation policies are concerned, relevant government agencies are inclined to offer subsidy funds to SMEs with large scale, low leverage, strong growth and certain independent characteristics of the board of directors. SMEs with strong growth often face financial pressure because of their rapid growth, which is not conducive to promoting innovation projects with higher risks and longer investment cycles [73], while the blessing of government subsidy funds is beneficial to “escort” the rapid growth of SMEs. As an important supervisory force of SME governance, independent directors play an important role in internal governance, and at the same time, they can express neutral opinions that are in line with the interests of small and medium shareholders on important decisions such as innovation. The higher the independence of the board of directors, the stronger the innovation ability of SMEs [74], which is conducive to improving the efficiency of government subsidies. However, different from government subsidies, relevant government agencies are willing to implement tax incentives for SMEs with weak growth. From the perspective of policy mix, the probability of SMEs receiving government innovation support is not only related to the scale and leverage ratio of SMEs, but also significantly affected by the ratio of assets expenditure. To be specific, the higher the asset expenditure ratio, the greater the possibility of government implementing policy mix to SMEs.

### 5.4 Moderating effect of region differences

To explore the moderating influence of regional disparities on SME innovation under distinct innovation policies, this section partitions the full sample into two subsets: the eastern region group (The eastern region includes 10 provinces, including Beijing, Tianjin, Hebei, Shandong, Jiangsu, Shanghai, Zhejiang, Fujian, Guangdong and Hainan.) and the central and western region group, based on the provinces where the SMEs are situated. The estimated outcomes for these two groups are presented in Table 6. In terms of “substantive innovation,” the impacts of innovation policies (government subsidy, tax incentive, and policy-mix) on SME innovation in the central and western region group surpass those in the eastern region group. Regarding “strategic innovation,” tax incentives and policy-mix exhibit heightened significance in fostering innovation among SMEs in central and western regions, while government subsidies prove more effective for SMEs in eastern regions. Overall, the policy-mix strategy proves more beneficial for advancing SME innovation, especially for SMEs located in eastern regions.

**Table 6:** Moderating effect of region differences.

Eastern region group (37951 observations)						
	Inno		Subs_inno		Stra_inno	
	ATT	S.E.	ATT	S.E.	ATT	S.E.
Sub	0.1439***	0.0144	0.0829***	0.0102	0.1044***	0.0124
Tax	0.1131***	0.0189	0.0776***	0.0136	0.0747***	0.0161
Mix	0.3209***	0.0171	0.1881***	0.0120	0.2304***	0.0143
Tax Vs. Sub	-0.0308	0.0217	-0.0053***	0.0019	-0.0297	0.0254
Mix Vs. Sub	0.1769***	0.0162	0.1052***	0.0133	0.1259***	0.0135
Mix Vs. Tax	0.2078***	0.0203	0.1105***	0.0143	0.1557***	0.0169
**Central and western region group (13211 observations)**						
	Inno		Subs_inno		Stra_inno	
	**ATT**	**S.E.**	**ATT**	**S.E.**	**ATT**	**S.E.**
Sub	0.4207***	0.0519	0.3525***	0.0266	0.0355	0.0206
Tax	0.2052***	0.0395	0.1105***	0.0267	0.1916***	0.0357
Mix	0.4177***	0.0296	0.2798***	0.0209	0.2626***	0.0273
Tax Vs. Sub	-0.2155	0.5208	-0.2120	0.4272	0.1561***	0.0353
Mix Vs. Sub	-0.0030	0.5895	-0.0727	0.4718	0.2271***	0.0267
Mix Vs. Tax	0.2125***	0.0429	0.1693***	0.0296	0.0710	0.0498

**Note:**

***,

**, and

*  represent the significance levels at 1%, 5%, and 10%, respectively; S.E. represents Standard Error.

### 5.5 Moderating effect of industry differences

To analyze the moderating impact of industry distinctions on SME innovation under various innovation policies, this segment categorizes the full sample into two subsets: the high-tech industry group and the non-high-tech industry group, in accordance with the National Bureau of Statistics GB/T4754 industry classification standard. The estimated results for these two groups are delineated in Table 7. The effect of innovation policies on SME innovation within the high-tech industry group appears to be less pronounced compared to SMEs in the non-high-tech industry group. To some extent, the impetus for innovation among high-tech SMEs may stem from industry competition, while non-high-tech SMEs draw their innovation drive from government policy support. Generally, the policy-mix approach consistently outperforms individual innovation policies in fostering SME innovation. It is noteworthy that for high-tech SMEs, government subsidies prove more effective than tax incentives; conversely, for non-high-tech SMEs, tax incentives play a more substantial role in stimulating innovation compared to government subsidies.

**Table 7:** Moderating effect of industry differences.

High-tech industry group (22205 observations)						
	Inno		Subs_inno		Stra_inno	
	ATT	S.E.	ATT	S.E.	ATT	S.E.
Sub	0.1241***	0.0189	0.0861***	0.0144	0.0892***	0.0161
Tax	0.0047	0.0239	0.0101	0.0179	0.0065	0.0201
Mix	0.1996***	0.0191	0.1286***	0.0145	0.1304***	0.0162
Tax Vs. Sub	-0.1193***	0.0222	-0.0761***	0.0165	-0.0826***	0.0187
Mix Vs. Sub	0.0756***	0.0168	0.0425***	0.0117	0.0412***	0.0115
Mix Vs. Tax	0.1949***	0.0223	0.1186***	0.0167	0.1238***	0.0188
**Non-high-tech industry group (28957 observations)**						
	Inno		Subs_inno		Stra_inno	
	**ATT**	**S.E.**	**ATT**	**S.E.**	**ATT**	**S.E.**
Sub	0.1389***	0.0187	0.0905***	0.0123	0.0938***	0.0158
Tax	0.2402***	0.0248	0.1334***	0.0167	0.1848***	0.0218
Mix	0.4631***	0.0406	0.2671***	0.0235	0.3404***	0.0267
Tax Vs. Sub	0.1013***	0.0216	0.0429***	0.0095	0. 0910***	0.0223
Mix Vs. Sub	0.3241***	0.0414	0.1766***	0.0242	0.2466***	0.0271
Mix Vs. Tax	0.2228***	0.0445	0.1336***	0.0267	0.1555***	0.0310

**Note:**

***,

**, and

*represent the significance levels at 1%, 5%, and 10%, respectively; S.E. represents Standard Error.

## 6 Conclusion and policy suggestions

This paper delves into China’s NEEQ listed enterprises spanning from 2011 to 2020 as the research sample, employing the Multi-Level Treatment Effect model to delve into the tangible impact of diverse innovation policies on SME innovation and the heterogeneity of policy effects through the lens of substantive and strategic innovation. Here are some pivotal findings outlined in this study. First, enhanced innovation through policies. Innovation policies notably enhance SME innovation, particularly substantive innovation. The efficacy of the policy-mix in stimulating SME innovation outweighs that of individual innovation policies. Second, selection criteria for policy favors. SMEs exhibiting “strong motivation” and “high ability” in innovation are more likely to garner favor from pertinent government bodies and possess a heightened likelihood of being the recipients of innovation policies. Third, regional policy optimalities. For SMEs situated in eastern regions, the policy-mix emerges as the optimal innovation policy, proficient in stimulating both substantive and strategic innovation. In contrast, SMEs in central and western regions may find government subsidies and policy-mix as the prime strategies for promoting substantive innovation, while tax incentives and policy-mix could be optimal for driving strategic innovation. Fourth, differential impact of single policies. Among single innovation policies, government subsidies prove more beneficial for high-tech SMEs compared to tax incentives, whereas non-high-tech SMEs witness a stronger impetus from tax incentives in fostering innovation over government subsidies.

This paper posits that innovation policies can exert a substantial influence on the innovation of SMEs. This finding aligns with the conclusions of previous studies [75–77], yet the present study goes beyond these earlier investigations. The present study delves into the innovation strategies employed by SMEs. The innovation of SMEs is divided into substantive innovation and strategic innovation, and the study finds that innovation policy has a more significant effect on promoting substantive innovation in SMEs. This phenomenon may be attributed to the fact that substantive innovation typically necessitates substantial technical support, financial resources for research and development, and skilled professionals. The implementation of innovation policies, such as financial subsidies and tax incentives, has been demonstrated to alleviate the constraints faced by SMEs in acquiring resources and developing technology. Building on the extant literature [78,79], this study aims to identify which SMEs are more likely to be affected by innovation policies. The findings of this study indicate that as the size of the SME increases and its leverage ratio decreases, there is a corresponding increase in the probability of the SME benefiting from innovation policy support. In light of the findings from this study, the following policy recommendations are put forward:

### (1) Tailored innovation policy support

Government entities should discern the unique attributes of diverse innovation entities and establish a multi-tiered innovation policy support framework. Given the evident regional and industrial discrepancies in the impacts of distinct innovation policies on fostering SME innovation, it is imperative for relevant government agencies to consider the balanced advancement of various regions and industries when executing and harmonizing the array of innovation policies. Special attention should be directed towards the central and western regions, characterized by underdeveloped and non-high-tech industries that stand to gain more from innovation backing.

### (2) Compr ehensive selection criteria

Relevant government bodies should holistically assess various factors influencing SME innovation and the likelihood of SMEs receiving governmental support. When pinpointing the beneficiaries of innovation policies, these agencies should take into consideration SME characteristics like enterprise scale, leverage ratio, enterprise growth, and the independence of the board of directors. Specifically, a focus on SME scale and leverage ratio is crucial when implementing a policy-mix.

### (3) Optimal policy combination

Government agencies should leverage the synergies and complementary nature of government subsidies and tax incentives to craft a rational policy-mix, thereby achieving the optimal fusion of innovation policies and innovation performance. Moreover, these agencies should actively incorporate and adopt innovative policies from other nations, devising new policy tools tailored to domestic innovation and development. By expanding the “toolbox” of innovation policy, offering a broader selection of policy combinations, and diversifying strategies, agencies can enhance the effectiveness of innovation policy optimization.

The study outlined certain limitations that could be addressed in future research to enhance the depth and breadth of the analysis. First, inclusion of management and marketing innovation. Future investigations could incorporate management innovation and marketing innovation within the research framework to provide a more holistic view of SME innovation dynamics. These dimensions are essential components of innovation and warrant closer examination to better capture the full spectrum of innovative activities within SMEs. Second, focus on innovation patent quality. Building on established practices in patent literature, future studies can shift their focus from the sheer count of patents to the quality of innovation patents. By utilizing patent citation data to gauge innovation patent quality, researchers can gain insights into the actual impact of policy-mix on the quality of innovation within SMEs. This shift can offer a more nuanced understanding of innovation outcomes and effectiveness. Third, exploration of additional innovation policies. While the current study primarily delves into the influence of government subsidies and tax incentives on SME innovation, future research can broaden its scope by incorporating additional innovation policies. By exploring policies such as government procurement and patent protection, researchers can create a more comprehensive understanding of the innovation policy landscape and its impact on SME innovation outcomes. This expansion can lead to a more realistic depiction of the interplay between various policy instruments and SME innovation performance.
